# Recombinant expression and characterization of two glycoside hydrolases from extreme alklinphilic bacterium *Cellulomonas bogoriensis* 69B4^T^

**DOI:** 10.1186/s13568-020-00979-8

**Published:** 2020-03-10

**Authors:** Fan Li, Jiaying Dong, Xue Lv, Yanqiu Wen, Shan Chen

**Affiliations:** School of Life Sciences, Northeast Normal University, Changchun, 130024 China

**Keywords:** Glycoside hydrolases, *Cellulomonas bogoriensis*, Alkaline thermo-tolerant, Salt-tolerant

## Abstract

Two novel glycoside hydrolases were cloned from the genomic DNA of alklinphilic bacterium *Cellulomonas bogoriensis* 69B4^T^ and functionally expressed in *Escherichia coli*. The two enzymes shared less than 73% of identities with other known glycosidases and belonged to glycoside hydrolase families 5 and 9. Recombinant Cel5A exhibited optimum activity at pH 5.0 and at a temperature of 70 °C, and Cel9A showed optimum activity at pH 7.0 and at a temperature of 60 °C. The two enzymes exhibited activity at alkaline pH 11 and were stable over a wide range of pH. The maximum activities of Cel5A and Cel9A were observed in 0.5 M NaCl and 1 M KCl, respectively. In addition, these two enzymes exhibited excellent halostability with residual activities of more than 70% after pre-incubation for 6 days in 5 M NaCl or 4 M KCl. Substrate specificity analysis revealed that Cel5A and Cel9A specifically cleaved the β-1,4-glycosidic linkage in cellulose with the highest activity on carboxymethyl cellulose sodium (78.3 and 145.3 U/mg, respectively). Cel5A is an endoglucanase, whereas Cel9A exhibits endo and exo activities. As alkali-activated, thermo-tolerant, and salt-tolerant cellulases, Cel5A and Cel9A are promising candidates for further research and industrial applications.

## Introduction

Cellulases are diverse enzymes that degrade cellulose by hydrolyzing the β-1,4 linkage that joins two glucose molecules (Wilson 2011). These enzymes include β-1,4-endoglucanase (EC 3.2.1.4), β-1,4-exoglucanase (EC 3.2.1.91), and β-1,4-glucosidase (EC 3.2.1.21) and can be grouped into many glycoside hydrolase families based on amino acid (aa) sequence (Nguyen et al. 2018). Cellulases have received extensive research attention because of their wide application potential in textile, detergent, pulp, feed, food, and biorefinery industries (Sharma et al. 2016). Despite the current long list of cellulases, only a few are considered feasible for wide-scale commercial applications due to the specific requirements in industries, such as superior stability at elevated temperature or at a certain pH and compatibility with surfactants, salts, and organic solutions. Therefore, novel cellulases that tolerate these adverse conditions must be exploited for application in biotechnology (Asha and Sakthivel 2014).

Extreme environment is a good source of enzymes with specific characteristics. Many cellulases with industrially relevant profiles were obtained from extremophiles (Ben Hmad and Gargouri 2017; Solingen et al. 2001; Susumu et al. 2002). Metagenomics technique has been recently applied to achieve a range of biocatalysts from these environments rich of potential uncultured extreme microorganisms (Ngara and Zhang 2018; Meilleur et al. 2009; Park et al. 2011; Pushpam et al. 2011).

*Cellulomonas bogoriensis* 69B4^T^ was isolated from the sediments and water of Lake Bogoria in Kenya with water temperature of 33 °C and pH of 10.5. Strain growth occurs at an optimum pH of approximately 9–10 and in medium containing 0–8.0% (w/v) NaCl (Jones et al. 2005). In a previous study, we purified an extracellular alkaline protease with optimum activity at pH 11 and stability at pH range of 3–12 from the strain (Li et al. 2016). We found that such strain can strongly degrade cellulose in an alkaline environment. In the present manuscript, we report the cellulase production of the strain and the gene cloning, expression, and characterization of the two glucanases from the strain.

## Materials and methods

### Bacterial strains, medium, and growth conditions

*Cellulomonas bogoriensis* 69B4^T^ (DSM 16987) was purchased from the German Collection of Microorganisms and Cell Cultures. Enzymes were produced using media containing 2.5% corn stalk, 1.0% beef extract, 1.0% yeast extract, 4% NaCl, 1% Na_2_CO_3_, 0.1% KH_2_PO_4_, and 0.02% MgSO_4_·7H_2_O at pH 10.6 and at a temperature of 30℃ for 72 h. In studying the effect of carbon source on enzyme production, the corn stalk in the medium was replaced with maltose, mannose, sucrose, starch, xylose, xylan, sorbose, corn stover, bran, carboxymethyl cellulose sodium (CMC), and cellulose CF-11. Similar effects of nitrogen sources, including NH_4_NO_3_, NH_4_Cl, (NH_4_)_2_SO_4_, NaNO_3_, urea, beef extract, peptone, yeast extract, and mixed organic nitrogen, were tested. Cellulase was produced by growing the bacterium in medium with different initial pH values from 6.6 to 11.6 and different culture temperatures from 20 to 42 °C.

### Enzyme assay

Culture supernatant or purified recombinase at appropriate concentration was mixed with CMC (1% w/v) substrate dissolved in Tris–HCl buffer (pH 8.6) at 50 °C for 20 min to assay the activity. The reducing sugar from CMC was measured by dinitrosalicylic acid method as described by (Miller 1959). One unit of CMCase was defined as the amount of enzyme required to release 1 µmol glucose per minute.

### Sequence analysis and gene cloning

Two cellulases were predicted in the genome sequence of *C. bogoriensis* 69B4^T^. Database homology search was performed using the NCBI BLAST program. Protein domains were predicted by BLAST searches against NCBI’s Conserved Domain Database. Multiple sequence alignments were performed using ClustalW (version 2) program (Larkin et al. 2007), and molecular mass and isoelectric point calculations were performed online by using an ExPASy-Protparam tool. Signal peptide sequence was predicted using SignalP 4.1 Server. The genes of *cel5A* and *cel9A* were amplified from the genomic DNA of *C. bogoriensis* 69B4^T^ through PCR method. The purified amplified products of *cel5A* and *cel9A* were doubled digested and subcloned into vector pET22b and pMAL-c2x, and then introduced into *Escherichia coli* DH5α for sequencing (Comate Bioscience, Changchun, China).

### Enzyme expression and purification

The plasmids containing the correct gene fragments were transformed into host strain *E. coli* Rosetta (DE3). The strains were cultured in LB medium (2% glucose is required for Cel9A recombinant strain) and induced by isopropyl β-d-1-thiogalactoside (IPTG) with a final concentration of 1 mM at 16 °C for 22 h. After centrifugation and washing, the harvested cells were suspended in lysis buffer (20 mM Tris–HCl buffer, pH 8.0) at 4 °C and lysed through sonication. The supernatant of Cel5A recombinant was applied to high-affinity Ni–NTA resin and purified through elution with imidazole gradient. Meanwhile, the supernatant of Cel9A recombinant was applied to amylose resin and purified through elution with 10 mM maltose.

### SDS-PAGE, native PAGE, and protein measurement

Protein was visualized by sodium dodecyl sulfate polyacrylamide gel electrophoresis (SDS-PAGE). The molecular weight of the purified enzyme was determined by comparing with a standard molecular weight marker in 10% SDS-PAGE gels. Native PAGE for in situ activity detection was performed according to the modified activity staining method using CMC as substrate (Schwarz et al. 1987). Protein concentration was quantified using Protein Assay Kits (KeyGEN, China) with bovine serum albumin as the standard.

### Effect of pH and temperature on the enzymes

The activity of the purified recombinase was assayed in 0.05 M buffers at various pH conditions containing citric acid buffer (pH 3.0–6.0), phosphate buffer (pH 6.0–8.0), Tris–HCl buffer (pH 8.0–9.0), and glycine–NaOH buffer (pH 9.0–13.0) to determine the optimum pH. The optimum temperature for the recombinase activity was determined through enzyme reaction from 10 to 80 °C. The enzyme was kept at a pH range from 3 to 13 at 4 °C for 24 h or at temperatures from 4 to 80 °C for 2 h to determine its pH stability and thermostability. Residual activity was assayed under the previously described conditions.

### Effect of metal ions, chemicals, and salts on the enzymes

Fe^2+^, Fe^3+^, Cu^2+^, Ca^2+^, Na^+^, K^+^, Ni^2+^, Mn^2+^, Co^2+^, Mg^2+^, and Zn^2+^ were added to the reaction at final concentrations of 1 and 10 mM to evaluate their effects on enzyme activity. Methanol, ethanol, glycerol, isopropanol, Triton-X 100, Tween-80, and SDS at final concentrations of 1% (v/v) and 10% (v/v) were added to the reaction to determine the effect of chemicals on the activity of the purified enzyme. The activity of the enzyme incubated without any metal ion and chemical was 100%.

Enzyme activity was analyzed in the reaction containing 0.5–4 M NaCl or KCl to detect the effect of salt concentration on the enzyme. For the measurement of halostability, the enzyme was incubated in buffer with 1–5 M NaCl or 1–4 M KCl for 1–7 days or 1–15 days at 4 °C. Residue activity was tested under optimal reaction conditions in 24 h intervals. The enzyme solution without any salt was set as the control with 100% activity.

### Substrate specificity

The substrate specificity of the enzyme was determined in different substrates containing CMC, beech xylan, Laminaria polysaccharide, filter paper (Whatman No. 1), Avicel, and corn stalk (1% w/v) for 20–120 min under optimum conditions.

### Kinetic analysis

Kinetic assays were performed under optimal temperature and pH conditions with CMC (0.5–10 mg/mL) as the substrate. Michaelis constant (*K*m) and maximum velocity (*V*max) were determined according to Lineweaver–Burk equation.

### Thin-layer chromatography (TLC) analysis

The degradation pattern of the enzyme on cellooligosaccharides (1%) was analyzed by TLC on Silica Gel 60 plates (Merck, Darmstadt, Germany). The plates were developed in chloroform/methanol/acetic acid/water (50:50:15:5, by vol). Sugars were visualized by spraying the plates with a freshly prepared mixture of ethanol/sulfuric acid (19:1, v/v), followed by heating at 110 °C for 5 min.

## Results

### Cellulase production by the strain

**Fig. 1:**
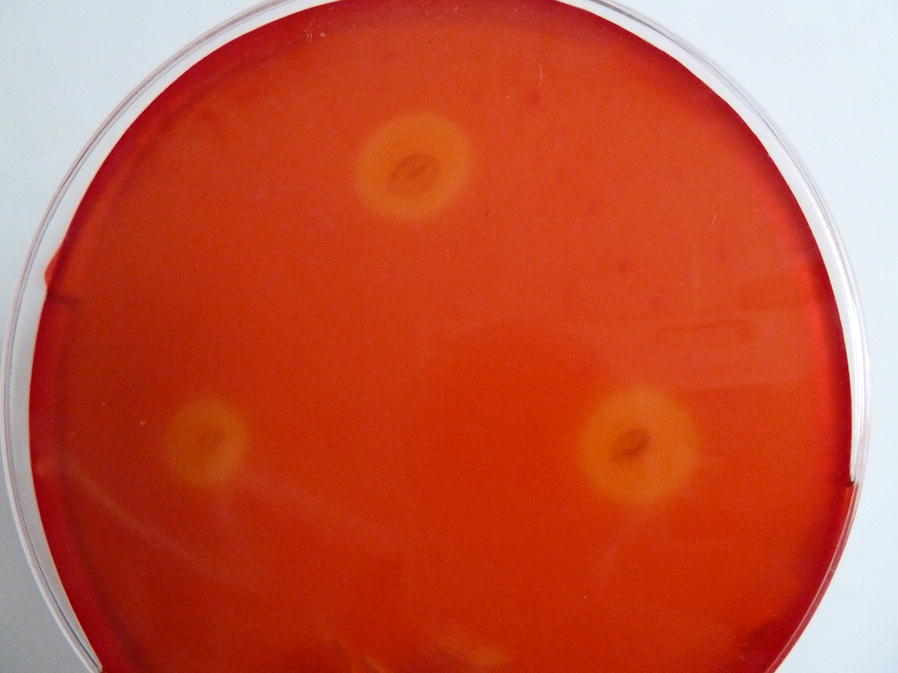
Clear-zones formed by *Cellulomonas bogoriensis* on congo red stained CMC agar medium

*Cellulomonas bogoriensis* 69B4^T^ efficiently degraded cellulose under alkaline conditions. After 2 days of strain growth on a CMC medium plate with pH 10.6, a clear hydrolytic zone was formed around the clone by congo red staining (Fig. 1). The effect of culture conditions, including carbon and nitrogen sources, initial pH, and temperature, on enzyme production was then investigated in the liquid culture. The results (Additional file 1: Figure S1) showed that natural composite carbon sources, such as corn stalk and bran, are beneficial for the production of cellulase, and organic nitrogen sources effectively promote enzyme production. Cellulase can be synthesized in the medium with initial pH from 6.6 to 11.6, and maximum cellulase was produced at pH 10.6. In terms of culture temperature, the strain is capable of producing cellulase in the range of 20 °C to 42 °C. Maximum enzyme activity was observed at 30 °C. Under these optimized conditions, the cellulase activity of the supernatant was 2.4 U/mL after 72 h of cultivation.

### Sequence analyses of Cel5A and Cel9A

Genomic sequence analysis showed that *Cel5A* and *Cel9A* (the genes were named on the basis of the characteristics of the family of glycoside hydrolases to which the catalytic domain belongs) may be related to cellulose degradation in the genome of *C. bogoriensis* 69B4^T^. *Cel5A* comprised an ORF of 1860 nucleotides encoded a protein of 619 aa, with a theoretical molecular weight of 68.05 kDa and a theoretical pI of 4.47. This protein was predicted to have a 20 aa signal peptide at the N-terminal region, a catalytic domain of glycoside hydrolase family 5 (GH5) located in 45–371 aa, and a cellulose binding domain CBM-3 in the C-terminal portion (474–556 aa). *Cel9A* comprised an ORF of 2403 bp encoded an 800-residue protein with theoretical molecular weight of 85.86 kDa and theoretical pI of 4.17. A 37 aa signal peptide was predicted at the N-terminal region of the protein. Different from Cel5A, this mature polypeptide contained a catalytic domain of glycoside hydrolase family 9 (GH9) (45–471 aa) linked to a family 3 CBM, followed by another binding domain CBM-2 at the C-terminal.

The alignment of Cel5A amino acid sequence with other protein sequences in GenBank revealed approximately 60% identity with some predicted cellulase family glycosyl hydrolases from *Streptomyces* sp. and 60.4% identity with endoglucanase Cel5B from *Thermobifida fusca* TM51. On the basis of sequence homologies with Cel5B, two conserved catalytic residues, Glu190 and Glu335, could be predicted as putative proton donor and nucleophile, respectively (Posta et al. 2004). Cel9A showed approximately 70% identity with some predicted endoglucanses from *Cellulomanas* sp. and 72.4% identity with endo/exocellulase E4 from *T. fusca.* Sequence alignment of Cel9A suggested that Glu462 was the acid that protonated the leaving group, and Asp58 was the base (Sakon et al. 1997). The multiple sequence alignments of Cel5A and Cel9A with other proteins are shown in Additional file 2: Figures S2 and Additional file 3: Figure S3. All comparison results indicated that Cel5A and Cel9A are novel endoglucanases that belong to families GH5 and GH9, respectively.

### Expression and purification of the recombinant enzymes

**Fig. 2:**
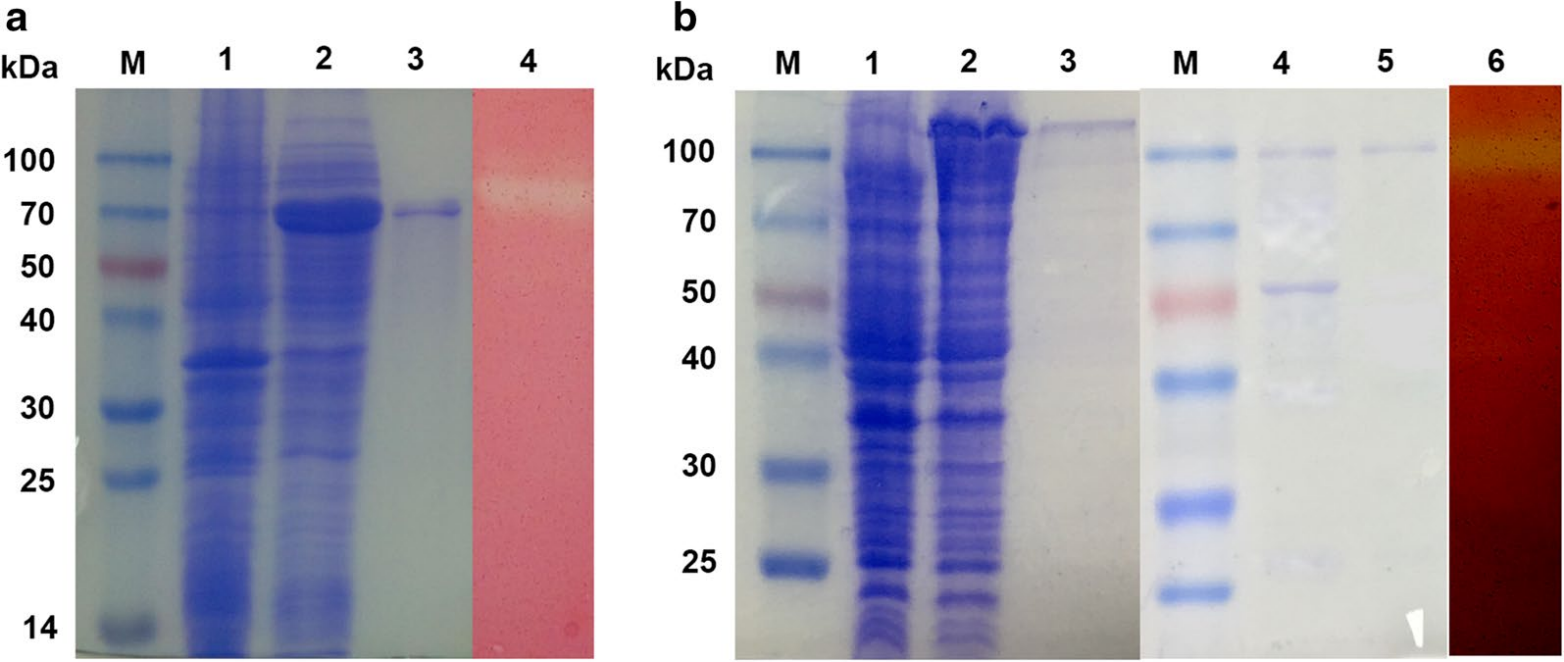
SDS-PAGE and zymogram of recombinant enzyme Cel5A (**a**) and Cel9A (**b**). Lane M molecular mass markers; **a** Lane 1 total protein of cells without induction, Lane 2 total protein of cells induced with IPTG, Lane 3 purified recombinant Cel5A, Lane 4 zymogram of purified Cel5A; **b** Lane 1 total protein of cells without induction, Lane 2 total protein of cells induced with IPTG, Lane 3 purified MBP fusion protein, Lane 4 fusion protein digestion by Factor Xa, Lane 5 purified recombinant Cel9A, Lane 6 zymogram of purified Cel9A

The cel5A gene, excluding the putative signal peptide sequence, was cloned as into vector pET22b and then expressed in *E. coli* Rosette (DE3). After purification by Ni–NTA affinity chromatography, Cel5A provided a single band with an apparent molecular mass of 66.5 kDa and zymogram activity on native-PAGE gel (Fig. 2a). A similar operation was performed for *Cel9A*, but the recombinant protein was expressed as inclusion body. Hence, *Cel9A* was cloned into the pMAL-c2x vector with a promoting fragment for expression. The recombinant Cel9A was purified from the cell lysate supernatant using amylose resin and then split by factor Xa to remove the fragment. SDS-PAGE analysis revealed a single band with an MW of 88 kDa for Cel9A close to the theoretical value, and the activity was observed on zymogram (Fig. 2b).

### Effect of pH and temperature on the recombinant enzymes

**Fig. 3:**
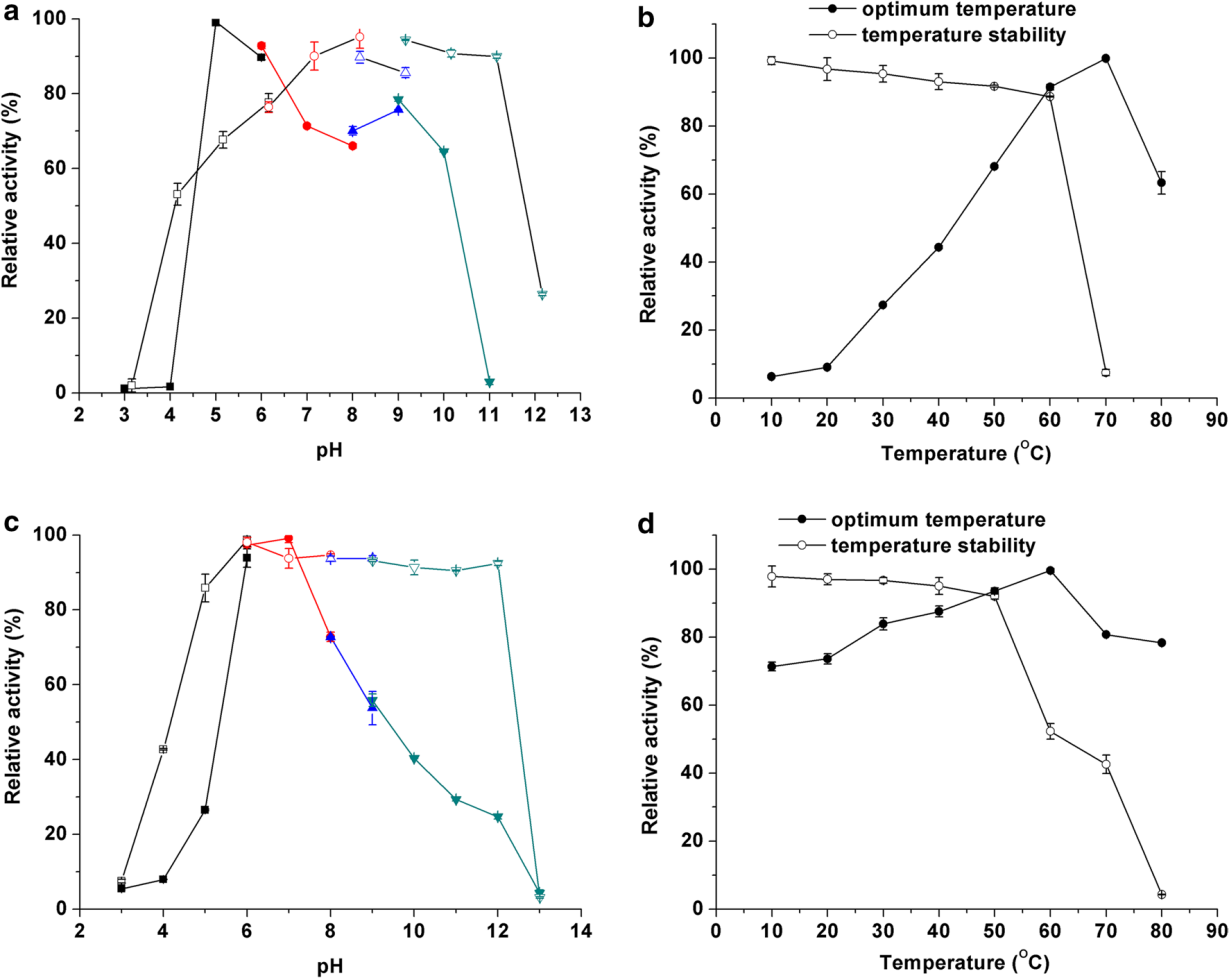
Effects of pH and temperature on the activity of Cel5A and Cel9A. **a** pH profile and pH stability of Cel5A, solid symbols represent the optimum pH and hollow symbols represent pH stability; **b** temperature dependence and temperature stability of Cel5A; **c** pH profile and pH stability of Cel9A, solid symbols represent the optimum pH and hollow symbols represent pH stability; **d** temperature dependence and temperature stability of Cel9A

The activity of purified enzymes was studied at different pH levels. The detected results of Cel5A showed the maximum activity at pH 5.0 in phosphate buffer and pH 9.0 in Gly-NaOH buffer; and the enzyme activity was greater than 60% in the pH range of 5–10 (Fig. 3a). Cel5A was also stable in a broad pH range and exhibited more than 70% activity from pH 5 to pH 11 (Fig. 3a). The activity profile of Cel9A showed that the highest activity occurred at pH 7.0, and it was reduced progressively with increasing pH from 7.0 to 12.0, which is more than 20% of the activity retained even at pH 12.0 (Fig. 3c). Cel9A also exhibited excellent pH stability with 80% of its maximum activity at a broad pH ranging from 5.0 to 12.0 (Fig. 3c). The effect of pH on the two enzymes indicated that they are cellulases with alkaline characteristics.

The effect of temperature on enzyme activity was determined at different temperatures ranging from 10 to 80 °C. The results showed that 70 and 60 °C were the optimal temperatures for Cel5A and Cel9A, respectively (Fig. 3b, d). These two enzymes also exhibited remarkable thermostability with more than 80% retained activity for Cel5A incubated at 60 °C for 2 h and approximately 40% retained activity for Cel9A incubated at 70 °C for 2 h (Fig. 3b, d). This profile with activity and stability at high temperatures is very advantageous for the application of alkaline enzymes in industries.

### Effect of metal ions and chemicals on the recombinant enzymes

The effects of various metal ions on the activities of Cel5A and Cel9A are listed in Table 1. The results showed that Ca^2+^, Ni^2+^, and Mg^2+^ enhanced the activity of Cel5A; Na^+^, K^+^, and Zn^2+^ exhibited no significant influence; Fe^3+^, Cu^2+^, Mn^2+^, and Co^2+^ inhibited the activity; whereas Fe^2+^ exhibited a distinctive effect with promoting activity at 1 mM and inhibiting action at 10 mM. The effect of most metal ions on Cel9A is similar to that on Cel5A, except for Cu^2+^, Ca2^+^, Ni^2+^, and Zn^2+^. These differences may be related to the different amino acid profiles that are important for catalysis or charge distribution.

**Table 1:** Effect of metals on recombinant enzymes

Metal ions	Relative activity (%)			
	Cel5A		Cel9A	
	1 mM	10 mM	1 mM	10 mM
Fe^2+^	132.5 ± 1.6	39.8 ± 6.0	129.0 ± 0.5	40.2 ± 7.1
Fe^3+^	75.8 ± 2.7	0	83.3 ± 1.7	21.0 ± 0.6
Cu^2+^	77.6 ± 0.4	45.4 ± 1.5	116.5 ± 0.5	35.3 ± 4.7
Ca^2+^	102.9 ± 2.2	113.5 ± 1.3	116.1 ± 1.5	71.7 ± 2.2
Na^+^	101.9 ± 1.1	101.8 ± 2.1	117.8 ± 1.5	102.3 ± 1.6
K^+^	103.7 ± 3.8	104.8 ± 0.3	105.6 ± 0.6	103.7 ± 1.7
Ni^2+^	107.64 ± 2.0	110.9 ± 1.6	81.5 ± 2.2	28.5 ± 4.1
Mn^2+^	71.3 ± 4.5	0	43.9 ± 4.3	56.8 ± 1.0
Co^2+^	75.5 ± 0.1	38.1 ± 1.5	76.0 ± 3.5	38.9 ± 6.3
Mg^2+^	103.3 ± 3.8	145.1 ± 0.4	95.3 ± 3.4	104.5 ± 1.8
Zn^2+^	93.4 ± 3.7	97.4 ± 2.6	72.6 ± 0.5	47.1 ± 2.0

Table 2 shows the effect of chemicals on the recombinant enzymes. The results indicated that Cel5A was tolerant to organic solvents with more than 80% activity in the presence of 1% and 10% methanol, ethanol, glycerin, and isopropanol. Surfactant Tween-80 and Triton X-100 exhibited no significant effect on the activity of Cel5A; whereas SDS reduced its activity to 4.6% at 10% concentration. Glycerin and Tween-80 stimulated the activity of Cel9A, whereas other chemicals remarkably detected inhibited Cel9A activity.

**Table 2:** Effect of chemical reagents on recombinant enzymes

Chemicals	Relative activity (%)			
	Cel5A		Cel9A	
	1%	10%	1%	10%
Methanol	92.7 + 3.8	87.5 + 0.6	98.9 ± 3.5	26.2 ± 3.4
Ethanol	96.7 + 1.6	86.1 + 2.3	73.4 ± 2.4	15.9 ± 0.71.7
Glycerin	87.5 + 0.9	86. 8 + 1.3	110.7 ± 1.7	111.3 ± 2.3
Isopropanol	93.7 + 0.3	87.4 + 0.1	118.7 ± 1.8	15.7 ± 1.9
SDS	66.3 + 0.4	4.6 + 0.8	26.3 ± 1.2	8.4 ± 1.7
Tween-80	100.1 + 0.5	102.6 + 2.6	101.7 ± 1.3	115.7 ± 1.8
Triton X-100	94.1 + 2.4	94.3 + 1.0	113.3 ± 4.0	63.5 ± 0.8

### Halotolerance of the recombinant enzymes

**Fig. 4:**
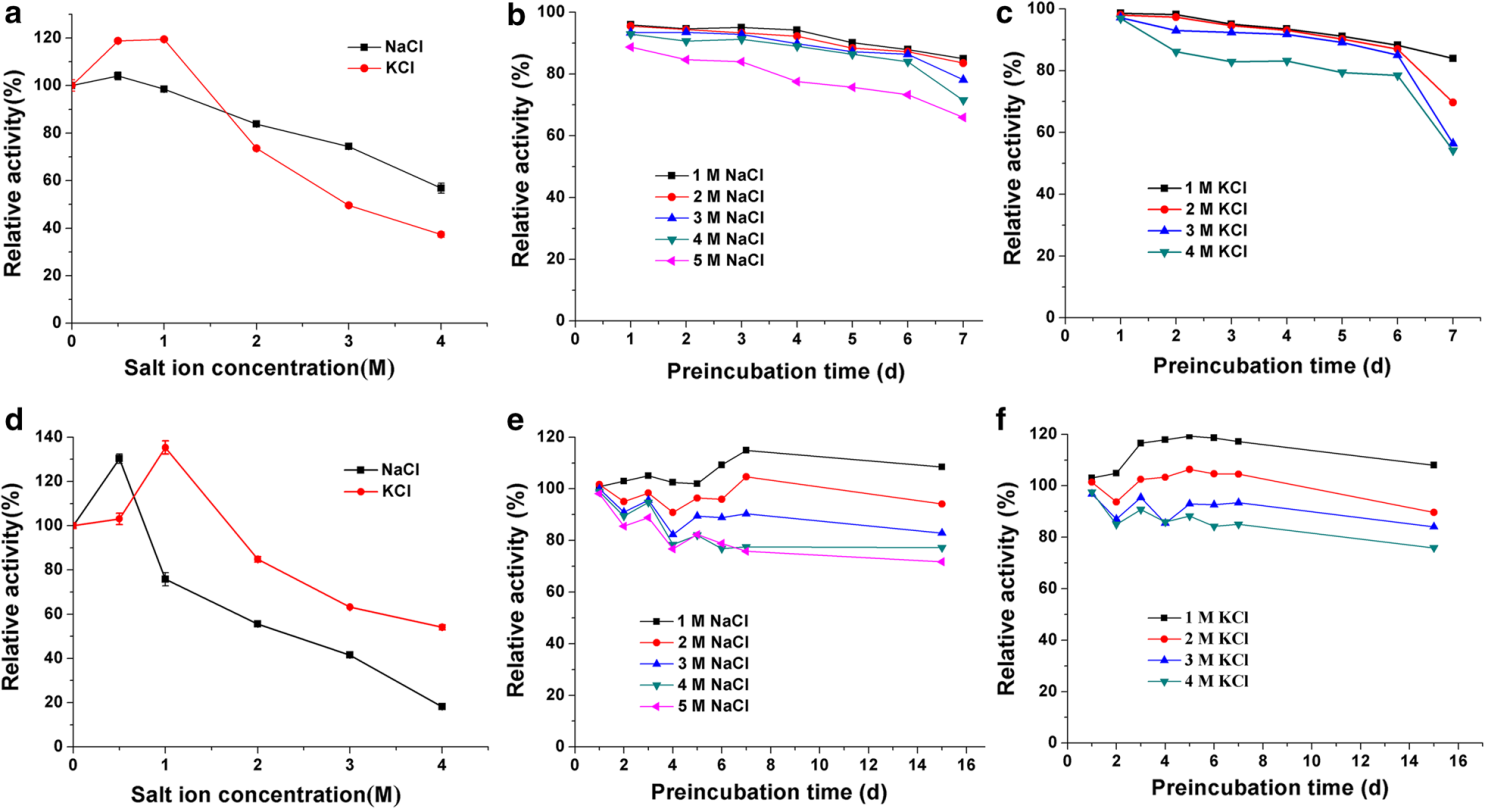
Effect of salts on the activity of of Cel5A and Cel9A. **a**, **d** Salt dependence of Cel5A and Cel9A; **b**, **e** halo-stability of Cel5A and Cel9A in NaCl; **c**, **f** halo-stability of Cel5A and Cel9A in KCl

Figure 4 shows the effect of salt on the activity of the recombinant enzymes. Salts with a concentration of less than 1 M stimulated enzyme activity, and the maximum activities of Cel5A and Cel9A were observed in 0.5 M NaCl or 1 M KCl (Fig. 4a, d). In addition, these two enzymes exhibited excellent halostability with more than 70% residual activities after pre-incubation for 6 days in 1–5 M NaCl or 1–4 M KCl, and the activity of Cel9A in 1–2 M NaCl/KCl even exceeded 100% (Fig. 4b, c, e, f). The high halotolerance of the enzyme is an important feature for its application in various biotechnological processes that depend on high salinity or osmotic pressure.

### Substrate specificity of the recombinant enzymes

Table 3 shows the substrate specificity of the purified Cel5A and Cel9A. Cel5A displayed major activity on soluble β-1,4-linked glucan CMC and less activities on crystalline cellulose, such as corn stalk, filter paper, and Avicel. No activity was observed against beech xylan and Laminarin. However, Cel9A has different substrate specificity with Cel5A. Cel9A showed high activities against CMC, beech xylan, and laminarin, indicating its broader substrate specificity for attacking β-1,3/4-linkage and β-1,3/6-linkage.

**Table 3:** Substrate specificity of the recombinant endogluanases

Substrate (1%)	Linkage	Solubility	Activity (U/mg)	
			Cel5A	Cel9A
CMC	β-1,4-linkage	soluble	78.3	145.3 + 0.8
Corn stalk	β-1,4-linkage	insoluble	3.0 + 0.5	0
Avicel	β-1,4-linkage	insoluble	15.7 + 1.4	20.0 + 1.8
Beech xylan	β-1,3/4-linkage	soluble	0	108.9 + 0.8
Laminarin	β-1,3/6-linkage	soluble	0	68.5 + 0.2
Filter paper	β-1,4-linkage	insoluble	1.6 + 0.4	7.0 + 0.4

### Kinetic analysis

Lineweaver–Burk curve was applied for catalytic kinetic analysis, and the kinetic parameters of the enzymes on CMC were calculated as described in the Materials and Methods. The *Km* and *Vmax* of Cel5A were 11.33 mg/mL and 166.67 µmol/min/mg, respectively; and for Cel9A, the corresponding values were 9.5 mg/mL and 304.87 µmol/min/mg, respectively.

### TLC analysis

**Fig. 5:**
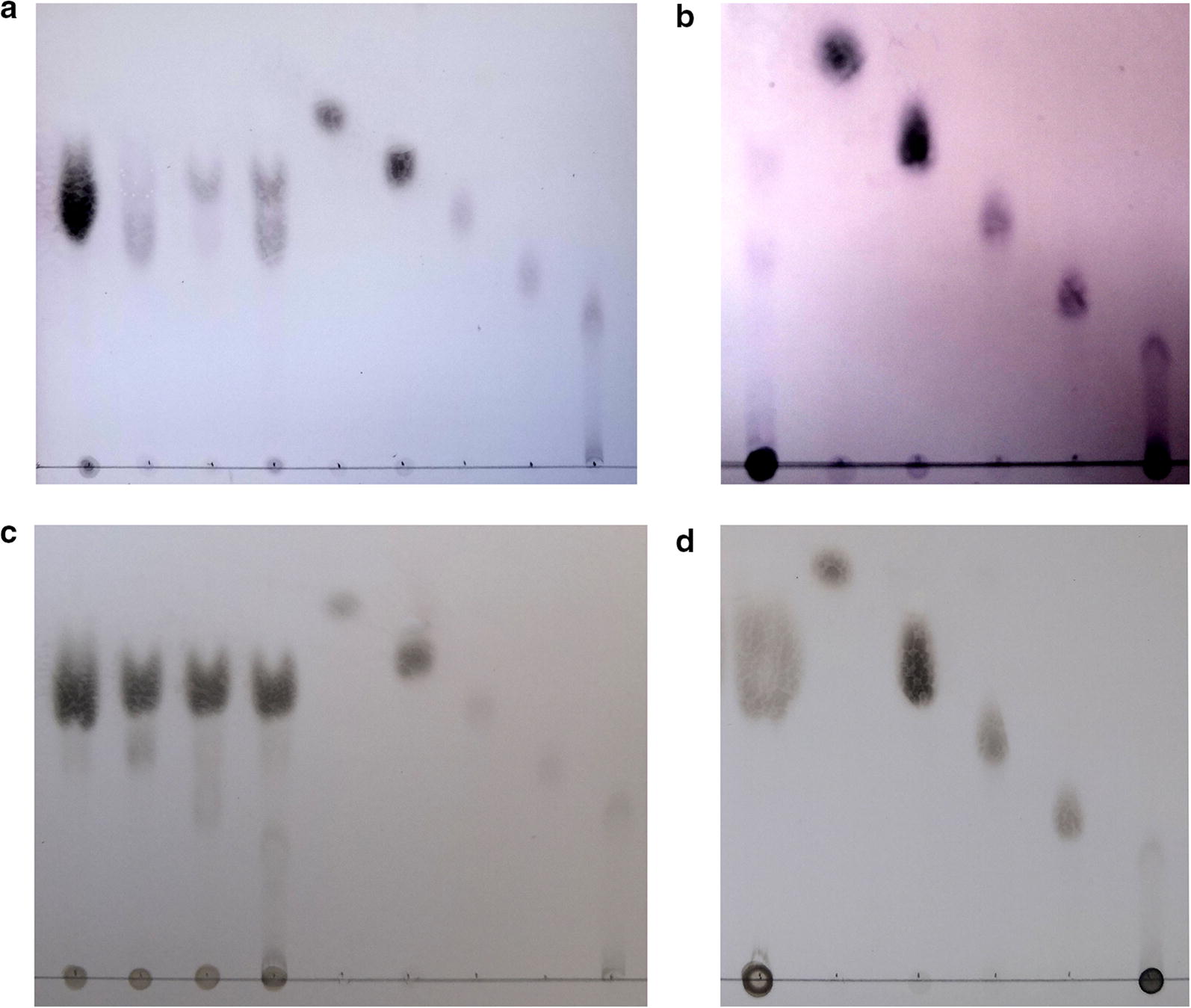
Thin-layer chromatography of the enzymatic hydrolysis product. Hydrolysis of cellooligomers by Cel5A (**a**) and Cel9A (**c**), the lanes from left to right are followed by cellobiose hydrolysate, cellotriose hydrolysate, cellotetraose hydrolysate, cellopentose hydrolysate, and standard glucose, cellobiose, cellotriose, cellotetraose and cellopentose. Hydrolysis of CMC by Cel5A (**b**) and Cel9A (**d**), the lanes from left to right are followed by CMC hydrolysate, glucose, cellobiose, cellotriose, cellotetraose and cellopentose

Cellooligomers and CMC were used as substrates to confirm the action mode of the enzyme. The TLC analysis (Fig. 5a) showed that no product was observed from G2 and G3 for Cel5A, and G4 was the smallest oligosaccharide that could be hydrolyzed with G2 as product. Degradation of CMC showed that cellobiose and cellooligosaccharides with high molecular mass were the main final products (Fig. 5b), indicating that Cel5A is an endo 1,4-glucan hydrolase. The product of Cel9A was also analyzed by TLC, and cellobiose was detected as the main product of enzymatic hydrolysis regardless of whether the substrate was cellooligosaccharide or CMC. Combined with the result of substrate specificity, Cel9A was considered to demonstrate endo and exo glucanase activities, which was consistent with the profile of cellulase from *T. Fusca* (Sakon et al. 1997).

## Discussion

Sequence alignments showed that Cel5A and Cel9A have low identity to other sequences in the NCBI database (less than 65% and 75%, respectively), indicating that they are novel enzymes. We compared these two cellulases with the enzymes from GH5 and GH9 families, respectively, and found that although the identity is low, many key amino acid residues are conserved in the sequence. For Cel5A, in addition to Glu190 and Glu335, which are identified as proton donor and nucleophile in the double-displacement reaction mechanism, some amino acids located in the catalytic active cleft related to substrate binding, such as Arg83, His141, Asn189, His290, Tyr292, and Trp367, were also highly conserved with respect to the other GH5 enzymes (Additional file 2: Figure S2). GH9 enzymes cleaved cellulose with the inversion of configuration at the anomeric carbon. The mechanism requires an acid to protonate the glycosidic oxygen and a base to extract a proton from a nucleophilic water that attacks the anomeric carbon nomenclature (Koshland 2008). Glu462 and Asp95 are the acid and base in Cel9A, respectively. The hydrophobic face of each glucose unit interacts with an aromatic side chain on the active site cleft of the cellulose-binding enzymes (Rouvinen et al. 1990; Spezio et al. 1993). Sequence alignments showed that the conserved residues, Trp294, Trp247, Trp351, Tyr244, Tyr458, Trp165 and His162 provide hydrophobic surfaces for interacting with Glc (−4)–Glc(+3). In addition to the above mentioned planar residues, some polar amino acids lining the active site cleft interacted with the glucosyl units proposed in E4 (Sakon et al. 1997), concluding that Asp92, Asp 95, Arg416, His414, Glu462 are all conserved in Cel9A.

The optimum pH values of Cel5A and Cel9A were 5 and 7, respectively, which are not within the alkaline range; but the enzymes exhibited a wide pH range. Cel5A demonstrated more than 60% activity in the range of pH 5 to 10. Cel9A has more than 50% enzyme activity at alkaline pH of 9 and 20% activity at pH of 12, indicating that these two enzymes have alkaline resistance. Cel5A is more alkaline than alkalophilic cellulase from *Bacillus vallismortis* RG-07 (Gaur and Tiwari 2015), cellulase from *Bacillus subtilis* SU40 (Asha and Sakthivel 2014), and endocellulase from *Streptomycete* sp. 11AG8 (Solingen et al. 2001). Another significant property of these two enzymes is their stability under alkaline conditions. Cel5A and Cel9A are quite stable under neutral and alkaline conditions from pH 6 to pH 11 and pH 5 to pH 12, respectively, suggesting that they are alkali resistant. The enzymes also exhibited other good properties, such as thermo-tolerant, and halo-tolerant, demonstrating their potential for specific industrial applications. At present, no definitive conclusion has been made on the alkaline adaptation mechanism of cellulase. Shirai et al. studied the structure and phylogenetic profiles of the alkaline cellulase K and found that Lys–Asp ion pairs are disfavored and partly replaced with Arg–Asp ion pairs in CelK, suggesting that the alkaline adaptation appeared to be a remodeling of ion pairs so that the charge balance remained at high pH (Shirai et al. 2001). The sequence analysis of Cel5A and Cel9A showed that the decrease of Lys and increase of Arg indicated that the charge balance caused by ion pairs may also be responsible for the alkaline resistance of the enzymes. The structure–function relationship of the enzymes must be further investigated.

Environmental stresses, such as extreme acidity or salinity, can bias the amino acid composition of species due to their desired chemical characteristics (Goodarzi et al. 2008; Paul et al. 2008). Paul et al. (2008) investigated the molecular signature of hypersaline adaptation from the genome and proteome composition of halophilic prokaryotes and proposed that halophilic species are characterized by low hydrophobicity, over-representation of acidic residues, especially Asp, lower propensities for helix formation, and higher propensities for coil (Paul et al. 2008). In our study, the hydrophobicity of Cel5A and Cel9A is − 0.433 and − 0.420 (Kyte and Doolittle 1982), respectively, in the hydrophobic range of halophilic microorganisms reported by Paul et al. Moreover, the isoelectric points of the two enzymes are 4.47 and 4.17, respectively, which are also consistent with the PI range of the proteins reported in the literature. In addition, the acidic amino acid content in Cel5A and Cel9A is significantly higher, and the ratios of acidic amino acids to basic amino acids are 1.55 and 2.08, respectively, which are higher than that of the endoglucanase from *T. fusca* TM51 (1.0) and close to that of Thcel6A from *Thermobifida halotolerans* (1.8) (Yin et al. 2015). These results indicate that the halophilic profile of the extracellular glycoside hydrolase derived from the extreme microbe is also associated with these molecular features derived from halophilic species and environments (Paul et al. 2008; Rhodes et al. 2010). These features can be studied for the screening of halophilic enzymes or the development of enzymes with extreme characteristics using protein engineering.

Most of the alkaline cellulases reported to date are mesophilic enzymes without thermo-tolerant characteristics and are therefore not suitable for many industrial applications that require high temperatures (Annamalai et al. 2011; Asha and Sakthivel 2014; Hakamada et al. 1997; Solingen et al. 2001; Yin et al. 2015). In recent years, few reports have been made on enzymes that have alkaline and thermostable properties, such as Cel5A from *Thermoanaerobacter tengcongensis* MB4, cellulase from *Bacillus halodurans* CAS 1, and cellulase from *Bacillus vallismortis* RG-07 (Annamalai et al. 2013; Gaur and Tiwari 2015; Liang et al. 2011). The two alkaline cellulases studied in this paper are thermo-tolerant and thermostable. The optimum temperature of Cel5A is 70 °C, and the activity remained more than 60% at 80 °C. After 2 h of incubation at 60 °C, the residue activity of the enzyme remained above 90%. Furthermore, Cel9A is an interesting full-temperature enzyme with more than 70% activity at a temperature range of 10–80 °C. It exhibited residual activity of more than 40% after incubation at 70 °C for 2 h. This thermo-tolerant characteristic makes them more robust in tough industry environment. In addition, although *C. bogoriensis* 69B4^T^ is not a thermophilic bacterium screened from high-temperature environments, such as hot springs, the amino acid sequences of Cel5A and Cel9A have a certain identity with that of cellulase derived from thermophilic microbe *Pyrococcus horikoshii* OT3 and *T. fusca*. Cel5A and Cel9A can be used as model enzymes to study the relationship between thermo-related properties and the environment and the evolution and migration of thermophilic cellulases in different microorganisms.

## Supplementary information

**Additional file 1: Figure S1.** Effect of culture conditions on cellulase production from *Cellulomonas bogoriensis*. (a) Effect of carbon source on cellulase production; (b) Effect of nitrogen source on cellulase production (1 NH_4_NO_3_, 2 NH_4_Cl, 3 (NH_4_)_2_SO_4_, 4 NaNO_3_, 5 urea, 6 beef extract, 7 peptone, 8 yeast powder, 9 peptone and beef extract (1:1), 10 peptone and yeast powder (1:1), 11 yeast powder and beef extract (1:1)); (c) Effect of initial pH on cellulase production; (d) Effect of temperature on cellulase production.

**Additional file 2: Figure S2.** Sequence alignment of the catalytic modules of Cel5A. Hypothetical Endo-1,4-beta-glucanase from *Pyrococcus horikoshii* OT3 (PDB no.2ZUM_A); Endocellulase E1 From *A. Cellulolyticus* (PDB no. 1VRX_A); Conserved residues are indicated by arrows.

**Additional file 3: Figure S3.** Sequence alignment of the catalytic modules of Cel9A. Endoglucanase 1 from *Hungateiclostridium thermocellum* (PDB no. 2XFG_A); 1,4-beta-glucanase from *Caldicellulosiruptor bescii* (PDB no.4DOD_A); Endoexocellulase from *Thermobifida fusca* (PDB no.1JS4_A); Conserved residues are indicated by arrows and black solid box.

## Data Availability

The accession number in NCBI Genbank database for Cel5A and Cel9A of *Cellulomonas bogoriensis* 69B4^T^ used in this research are KGM13741.1 and KGM14108.1.
