# Supplementary material for: Recombinant expression and characterization of two glycoside hydrolases from extreme alklinphilic bacterium Cellulomonas bogoriensis 69B4T
Source: AMB Express. 2020 Mar 10;10:44. doi: 10.1186/s13568-020-00979-8 (PMC7064699; doi:10.1186/s13568-020-00979-8)
Supplement: Supplementary file 1 — Additional file 1: Figure S1. Effect of culture conditions on cellulase production from Cellulomonas bogoriensis. (a) Effect of carbon source on cellulase production; (b) Effect of nitrogen source on cellulase production (1 NH4NO3, 2 NH4Cl, 3 (NH4)2SO4, 4 NaNO3, 5 urea, 6 beef extract, 7 peptone, 8 yeast powder, 9 peptone and beef extract (1:1), 10 peptone and yeast powder (1:1), 11 yeast powder and beef extract (1:1)); (c) Effect of initial pH on cellulase production; (d) Effect of temperature on cellulase production. [file 13568_2020_979_MOESM1_ESM.pdf]

AMB Express Additional Material

Recombinant expression and characterization of two glycoside hydrolases from extreme alklinphilic bacterium *Cellulomonas bogoriensis* 69B4^{T}

Fan Li*, Jiaying Dong, Xue Lv, Yanqiu Wen, Shan Chen

School of Life Sciences, Northeast Normal University, Changchun, 130024, China.

*corresponding author, E-mail:lif885@nenu.edu.cn

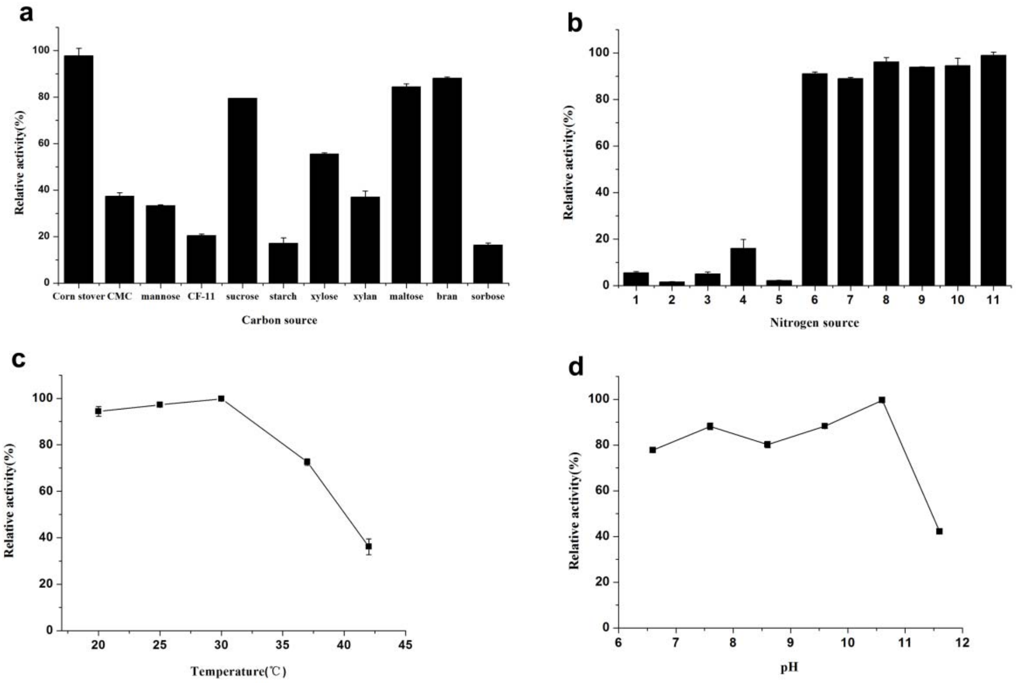

Fig S1. Effect of culture conditions on cellulase production from *Cellulomonas bogoriensis*. (a) Effect of carbon source on cellulase production; (b) Effect of nitrogen source on cellulase production (1 NH_4NO_3, 2 NH_4Cl, 3 $(NH_4)_2SO_4$, 4 $NaNO_3$, 5 urea, 6 beef extract, 7 peptone, 8 yeast powder, 9 peptone and beef extract (1:1), 10 peptone and yeast powder (1:1), 11 yeast powder and beef extract (1:1); (c) Effect of initial pH on cellulase production; (d) Effect of temperature on cellulase production.
