# Supplementary material for: Recombinant expression and characterization of two glycoside hydrolases from extreme alklinphilic bacterium Cellulomonas bogoriensis 69B4T
Source: AMB Express. 2020 Mar 10;10:44. doi: 10.1186/s13568-020-00979-8 (PMC7064699; doi:10.1186/s13568-020-00979-8)
Supplement: Supplementary file 2 — Additional file 2: Figure S2. Sequence alignment of the catalytic modules of Cel5A. Hypothetical Endo-1,4-beta-glucanase from Pyrococcus horikoshii OT3 (PDB no.2ZUM_A); Endocellulase E1 From A. Cellulolyticus (PDB no. 1VRX_A); Conserved residues are indicated by arrows. [file 13568_2020_979_MOESM2_ESM.pdf]

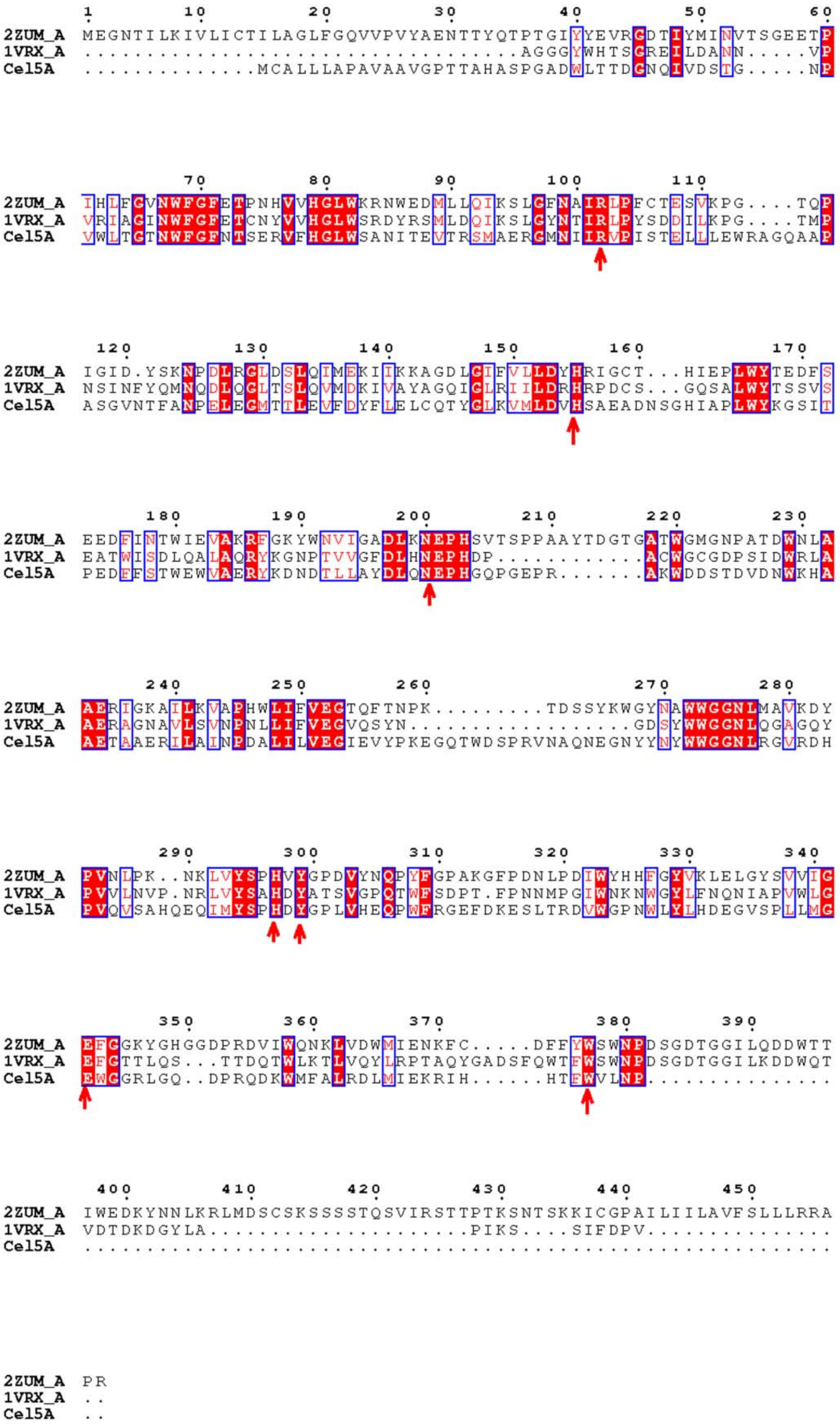

Fig S2 Sequence alignment of the catalytic modules of Cel5A. Hypothetical Endo-1,4-beta-glucanase from *Pyrococcus horikoshii* OT3 (PDB no.2ZUM_A); Endocellulase E1 From *A. Cellulolyticus* (PDB no. 1VRX_A); Conserved residues are indicated by arrows.
