# Supplementary material for: Recombinant expression and characterization of two glycoside hydrolases from extreme alklinphilic bacterium Cellulomonas bogoriensis 69B4T
Source: AMB Express. 2020 Mar 10;10:44. doi: 10.1186/s13568-020-00979-8 (PMC7064699; doi:10.1186/s13568-020-00979-8)
Supplement: Supplementary file 3 — Additional file 3: Figure S3. Sequence alignment of the catalytic modules of Cel9A. Endoglucanase 1 from Hungateiclostridium thermocellum (PDB no. 2XFG_A); 1,4-beta-glucanase from Caldicellulosiruptor bescii (PDB no.4DOD_A); Endoexocellulase from Thermobifida fusca (PDB no.1JS4_A); Conserved residues are indicated by arrows and black solid box. [file 13568_2020_979_MOESM3_ESM.pdf]

```
                        1         10                20            30             40
2XFG    ...............MENKNGGYLPEEEIPDQ..PPAXGAFNYGEALQKAIFFYECQRSG
4DOD    ...............MGSSHHHHHHSSGLVPRGSHMASGSFNYGEALQKAIMFYEFQMSG
Cel9A   MTRPAPPRRRLVAWGAGAAVVAGALGTAPLTAAPAAAEPSYNYGEALQKSMFFYEAQRSG
1JS4    .....................................EPAFNYAEALQKSMFFYEAQRSG

              50            60            70            80            90           100
2XFG    KLDSSTLRLNWRGDSGLDDGKDAGIDLTGGWYDAGDHVKFNLPMSYSAAMLGWAVYEYED
4DOD    KLPN.WVRNNWRGDSALKDGQDNGLDLTGGWFDAGDHVKFNLPMSYTGTMLSWAVYEYKD
Cel9A   PLPE.DNRVSWRGDSALDDGSDVGVDLTGGWYDAGDHVKFGFPMAFTATMLTWGALAYPD
1JS4    KLPE.NNRVSWRGDSGLNDGADVGLDLTGGWYDAGDHVKFGFPMAFTATMLAWGAIESPE

             110           120           130           140           150           160
2XFG    AFKQSGQYNHILNNIKWACDYFIKCHPEKDVYYYQVGDGHADHAWWGPAEVMPMERPSYK
4DOD    AFVKSGQLEHILNQIEWVNDYFVKCHPSKYVYYYQVGDGSKDHAWWGPAEVMQMERPSFK
Cel9A   GYEQSGQMPYLKDNIRWVNDYFLKAHTAPNELWVQVGDGDEDHKWWGPAEVMTMDRPSAK
1JS4    GYIRSGQMPYLKDNLRWVNDYFIKAHPSPNVLYVQVGDGDADHKWWGPAEVMPMERPSFK

             170           180           190           200           210           220
2XFG    VDRSSPGSTVVAETSAALAIASIIFKKVDGEYSKECLKHAKELFEFADTTK..SDDGYTA
4DOD    VTQSSPGSTVVAETAASLAAASIVLKDRNPTKAATYLQHAKELYEFAEVTK..SDAGYTA
Cel9A   IDPSCPGSDVAAETAAAMATASMLFADDDPAYSARLLESAVQLYDFADTYRGAYSDCLTQ
1JS4    VDPSCPGSDVAAETAAAMAASSIVFADDDPAYAATLVQHAKQLYTFADTYRGVYSDCVP.

               230           240           250           260           270           280
2XFG    ANGFYNSWSGFYDELSWAAVWLYLATNDSSYLDKAESYSDKWGYEPQTNIPKYKWAQCWD
4DOD    ANGYYNSWSGFYDELSWAAVWLYLATNDSTYLTKAESYVQNWPKISGSNTIDYKWAHCWD
Cel9A   VSPFYRSWSGYQDELVWGAYWLYEATGDEAYLAKAEAEYQYLGTENQTDTRSYRWTVNWD
1JS4    AGAFYNSWSGYQDELVWGAYWLYKATGDDSYLAKAEYEYDFLSTEQQTDLRSYRWTIAWD

               290           300           310           320           330           340
2XFG    DVTYGTYLLLARIKNDNGKYKEAIERHLDWWTTGYNGERITYTPKGLAWLDQWGSLRYAT
4DOD    DVHNGAALLLAKITG.KDIYKQIIESHLDYWTTGYNGERIKYTPKGLAWLDQWGSLRYAT
Cel9A   DKSFATYPLLAMATG.NQEYIDDTNRWLDFWTSGYDGQRVAYSPGGMAVLDSWGALRYAA
1JS4    DKSYGTYVLLAKETG.KQKYIDDANRWLDYWTVGVNGQRVPYSPGGMAVLDTWGALRYAA

               350           360           370           380           390
2XFG    TTAFLACVYSDWENGDKEKAKTYLEFARSQADYALGSTGR..SFVVGFGENPPKRPHHRT
4DOD    TTAFLAFVYSDWVGCPSTKKEIYRKFGESQIDYALGSAGR..SFVVGFGTNPPKRPHHRT
Cel9A   NTAFVALVYSDWIEDD.TRSARYHDFAVSQIDYALGDNPRSSSYVVGFGANPPQHPHHRT
1JS4    NTAFVALVYAKVIDDP.VRKQRYHDFAVRQINYALGDNPRNSSYVVGFGNNPPRNPHHRT

       400           410           420           430           440           450
2XFG    AHGSWADSQMEPPEHRHVLYGALVGGPDS.TDNYTDDISNYTCNEVACDYNAGFVGLLAK
4DOD    AHSSWADSQSIPSYHRHTLYGALVGGPGS.DDSYTDDISNYVNNEVACDYNAGFVGALAK
Cel9A   AHGSWLDSLTQPVETRHVLYGALVGGPPSPNDSYTDDRSDYVANEVAIDYNAGF......
1JS4    AHGSWTDSIASPAENRHVLYGALVGGPGSPNDAYTDDRQDYVANEVATDYNAGFSSALAM

      460
2XFG    MYKLYGEL....................................................
4DOD    MYQLYGGNPIPDFKAIE...........................................
Cel9A   ............................................................
1JS4    LVEEYGGTPLADFPPTEEPDGPEIFVEAQINTPGTTFTEIKAMIRNQSGWPARMLDKGTF

2XFG    ..
4DOD    ..
Cel9A   ..
1JS4    RY
```

Fig S3 Sequence alignment of the catalytic modules of Cel9A. Endoglucanase 1 from *Hungateiclostridium thermocellum* (PDB no. 2XFG_A); 1,4-beta-glucanase from *Caldicellulosiruptor bescii* (PDB no.4DOD_A); Endoexocellulase from *Thermobifida fusca* (PDB no.1JS4_A); Conserved residues are indicated by arrows and black solid box.
